# Multi-Residential Activity Labelling in Smart Homes with Wearable Tags Using BLE Technology

**DOI:** 10.3390/s18030908

**Published:** 2018-03-19

**Authors:** Ghassem Mokhtari, Amjad Anvari-Moghaddam, Qing Zhang, Mohanraj Karunanithi

**Affiliations:** 1 Deloitte Consulting Pty Ltd., Riverside Center, Brisbane 4000, Australia; amokhtari@deloitte.com.au; 2 CSIRO Australian e-Health Research Center, Butterfield St & Bowen Bridge Rd, Herston, QLD 4029, Australia; mohan.karunanithi@csiro.au; 3 Department of Energy Technology, Aalborg University, 9220 Aalborg East, Denmark; aam@et.aau.dk

**Keywords:** smart home, activity labelling, BLE, multi-residency, embedded system, wearable tags

## Abstract

Smart home platforms show promising outcomes to provide a better quality of life for residents in their homes. One of the main challenges that exists with these platforms in multi-residential houses is activity labeling. As most of the activity sensors do not provide any information regarding the identity of the person who triggers them, it is difficult to label the sensor events in multi-residential smart homes. To deal with this challenge, individual localization in different areas can be a promising solution. The localization information can be used to automatically label the activity sensor data to individuals. Bluetooth low energy (BLE) is a promising technology for this application due to how easy it is to implement and its low energy footprint. In this approach, individuals wear a tag that broadcasts its unique identity (ID) in certain time intervals, while fixed scanners listen to the broadcasting packet to localize the tag and the individual. However, the localization accuracy of this method depends greatly on different settings of broadcasting signal strength, and the time interval of BLE tags. To achieve the best localization accuracy, this paper studies the impacts of different advertising time intervals and power levels, and proposes an efficient and applicable algorithm to select optimal value settings of BLE sensors. Moreover, it proposes an automatic activity labeling method, through integrating BLE localization information and ambient sensor data. The applicability and effectiveness of the proposed structure is also demonstrated in a real multi-resident smart home scenario.

## 1. Introduction

The digital technologies that are deployed in smart home environments provide a lot of benefits for the residents, including efficiency and comfort enhancement [1,2]. The main objective of this platform is automation and energy management [3,4]; however, it can also provide different services for other applications, such as health and security. This platform uses different wireless sensors (that are mainly installed in the house), such as motion, power, humidity, and temperature, to continuously monitor residents and record their events. These recorded raw data will be used to recognize the residents’ activities and detect any abnormal event that requires further actions [5].

There are several challenges in the activity recognition research area that are still the focus of researchers around the world. One of the main challenges that exists includes activity recognition in a smart home with multiple residents. When there is only one resident, all of the recognized activities can be easily assigned to a certain profile. However, when there are multiple residents living in the same home, their activities cannot be properly recognized and separated, as the sensors’ data do not provide any information about the identity of the person who triggers the sensor. Therefore, to have accurate activity recognition in a smart home with multiple residents, an approach is needed to distinguish between different residents, while helping to label the sensor data. Individual localization and identification technologies are suitable options to be applied for this purpose.

Several types of approaches that can provide identity (ID) information are introduced in the literature. These approaches include the following:

Vision-based approach: This approach uses cameras to monitor multiple residents while recognizing their activities [6]. Biometric information, such as height and face, are the features that are used to identify multiple residents. The main issue with this approach is privacy.

Signature-based approach: In this type of approach, technologies such as microphone [7], passive infrared (PIR) [8], ultrasound [9], and ultra-wide band (UWB) [10] are used to generate a signature for residents. It is shown that this signature is unique and can be used to distinguish between residents while they are moving around the house. The accuracy and capability of this approach in a real smart home is still the focus of researchers’ attention.

Tag-based approach: In this approach, targets carry a tag as a unique ID, which uses different communication technologies, such as radio-frequency identification (RFID) [11,12,13], Bluetooth [14,15,16,17,18,19,20], ZigBee, and Wi-Fi [21,22,23,24], to broadcast their unique ID in a specific time interval. Additionally, there are scanners allocated in different areas that listen to the broadcasting packets to localize the tag and the individual.

For the application of these technologies in smart homes, the authors of [11] use an RFID tag as the unique ID for the resident, while a received signal strength indication (*RSSI*) index is used to find the resident’s location in a smart home. In a similar fashion, the authors of [12] present the latest research progress in an effort to include RFID antenna gain patterns in model-based received signal strength (RSS) localization algorithms. In the same work, it is shown that real-world, non-isotropic gain patterns of RFID badges are not a problem to be ignored, but a means to improve localization accuracy. A demo on fine-grained RFID localization via UWB emulation is presented in [13], where the proposed system allows users to move RFID-tagged objects to any location in line-of-sight, non-line-of-sight, and multi-path rich settings, and check that the system can accurately localize the objects. Another tag-based approach—based on the use of emerging Bluetooth low energy (BLE) devices (beacons), coupled with a mobile app and its intrinsic features—is outlined in [15]. It is recommended by the authors that to ease the localization, appropriate filtering or data processing after the initial distance estimation should be carried out. The researchers in [16] propose a hybrid real-time indoor localization architecture, based on smartphone and BLE beacons exploiting the complementary characteristics of the proximity (RSS method) and pedestrian dead-reckoning (PDR) approaches. Likewise, the authors of [17] report the design, implementation, and evaluation of an Internet of Things (IoT)-based system for indoor localization using BLE technology. Although the proposed approach could estimate the position with good precision, it requires a long training process and the use of different machine learning classifiers, which makes it inefficient for application in big office halls or larger areas. The performance of BLE technology for indoor positioning systems in different transmission conditions is also discussed in [18], where the localization method is implemented based on the *RSSI* value. In the same subject area, the researchers in [19] propose a method to render indoor localization and tracking practical, using only BLE sensors. In the same work, a distribution of *RSSI* values (fingerprint measurements) for every position inside the region of interest is estimated, and a direct relationship between the accuracy of the radio map and the accuracy of localization and tracking is extracted. However, as in the proposed approach, initial scene analysis is a must because it is not practical to collect data from a high number of locations.

The authors of [23] use the smartphone and its Bluetooth technology as the tag for the resident. The work also proposes that Bluetooth should be used rather than Wi-Fi, as it can provide good accuracy for room-level localization because of its limited energy level, which is usually limited to within a single room. On the other hand, the authors of [24] present a positioning method with special wireless signal module configuration rules to reduce the complexity of the calculation process, and the running time. However, the proposed approach suffers from an error that is caused by the energy error due to the distant nodes’ positions.

As can be understood from the reviewed literature, there are plenty of methods and technologies to detect and monitor devices/people in an indoor environment, and each of them has its own pros and cons. However, in the case of healthcare systems, especially those applied for aged care, it is recommended to use technologies that fulfill requirements such as low cost, low power, high accuracy and reliability, real time operation, and good resistance to fading (caused by multipath propagation) [25]. Based on these requirements, BLE is one of the promising technologies.

In this paper, BLE devices are used as the tag for multiple residents to localize and label their activities in a smart home. This solution not only provides an efficient way of transferring messages with low energy, but also gives high positioning accuracy, simple position estimation, and a lightweight and easily deployable tracking system. Compared to the reviewed literature, the contributions of this work can be summarized as follows:

- Room-level accuracy is considered as the required accuracy for localization in smart home applications. In this system, the resident is carrying a BLE tag as his/her identity, which broadcasts a unique ID in a specific time interval.
- To handle energy error issues, BLE scanners are optimally allocated in each room; they listen to the advertising packet and *RSSI* index to detect the presence of the resident in a given room.
- A comprehensive study is conducted to examine the impacts of broadcasting parameters on *RSSI* and to design a high accuracy localization approach.
- The possibility of knowledge extraction from other sensory networks for better localization is explored. As there are activity sensors installed in different rooms for the purpose of activity recognition, it is shown that the data from these sensors can also be used to improve the accuracy of localization and activity labeling.

## 2. Localization in Smart Homes Using BLE Technology

BLE technology is a promising communication technology for future smart homes because of its low energy consumption [26]. This technology, due to its low data rate and low energy consumption, is applied in several studies for the purpose of indoor localization. To this end, the researchers in [27] provide a comprehensive study on the performance of this technology for indoor localization, while the authors of [28] make a comparative study with Wi-Fi for the purpose of indoor localization.

**Figure 1:**
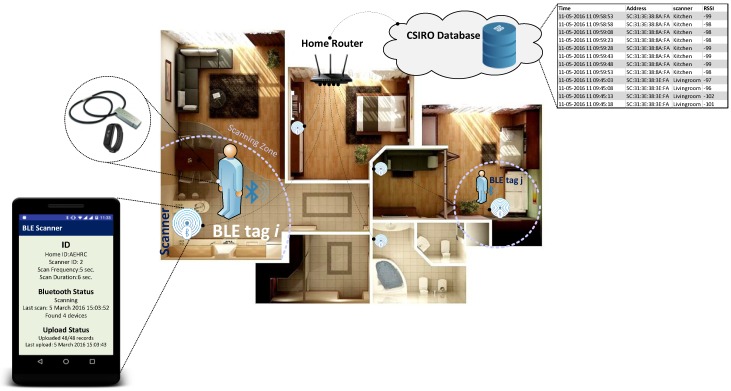
Implemented setup for multi-resident localization using Bluetooth low energy (BLE) technology.

Figure 1 shows the implemented structure of the multi-residential localization approach in the Commonwealth Scientific and Industrial Research Organization (CSIRO) smart safer home (SSH) platforms [29] using BLE technology. As shown in this figure, this structure includes two main parts. The first part includes BLE tags that are generic iBeacons carried by the residents. Tags have a MAC address as a unique ID, which is considered as the identity of the resident. Tags advertise this unique ID in a fixed time interval (advertising interval (*T_A_*)).

**Figure 2:**
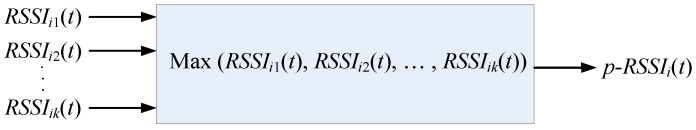
*p-RSSI* index.

The second part includes BLE Scanners, which are Android mobile phones in this case. As room-level accuracy is required, one scanner is considered for each room. A mobile application is developed, as the scanner, to listen to the BLE tags’ advertisements every second at the scanning time interval (*T_S_*). During the scanning duration, if there is any BLE tag that is in the range of a BLE scanner, it has the possibility of being detected. For instance, in Figure 2, BLE tag *i* has the possibility of being detected by the scanner, while BLE tag *j* cannot be detected. Beside this ranging criterion, there are other criteria that need to be met for the BLE tag to be detected by the scanner. The details of these criteria can be found in [14].

Scanners record the data of each BLE tag, including the time of scanning, tag ID (MAC address), and *RSSI*, and send them to the CSIRO database for further processing. This set-up is implemented as part of the SSH platform to see how these data, and data from activity sensors, can be used for accurate activity labelling of multiple residents.

The objective of this paper is to localize the residents at each time step of *T*. In other words, the aim is to localize the residents in different rooms during each time period (*T*), if they are at home. The value of *T* depends on the minimum time that a resident is in a room. For instance, if the individuals are likely to stay in a room for at least 30 s before transitioning to different rooms, the maximum time step *T* for the localization should be 30 s, in order to have an acceptable localization accuracy.

As the scanning zones of the scanners may interfere with each other, one tag may be scanned by two or more scanners in the same time step. *RSSI* will be used to associate the tag with the closest scanner and room. Additionally, as the advertising interval is usually less than *T*, the scanner may record more than one *RSSI* for each tag in each time step *T*. Therefore, to decide on the location of the tag, the representative *RSSI* value should be considered in each *T*. Two representative indices are used in this paper. The first index is named as *p*-*RSSI* index that considers the maximum *RSSI* as the representative *RSSI* of the tag scanned by the scanner in each *T*. If there are *k* recorded *RSSI* values for tag *i* in a given time *T*, then the representative *RSSI* for this tag is the maximum *RSSI* during this period, as given in Figure 2.

**Figure 3:**
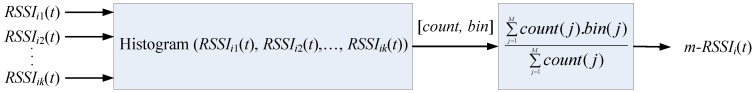
*m*-*RSSI* index.

The second index (which is named as *m*-*RSSI*) calculates the average of the *RSSI*s based on their histogram, as shown in Figure 3.

The algorithm used in the proposed structure includes two main parts to detect and localize each resident. The first part in this algorithm uses raw BLE data and the representative *RSSI* to localize the residents in each room during each *T*. This algorithm includes the following steps for each tag:

- Step 1: For each time interval *T*, all of the *RSSI* for the tag, scanned by different scanners, are recorded.
- Step 2: The representative *RSSI* in each scanner is determined based on either the *p*-*RSSI* or the *m*-*RSSI* model, shown in Figure 2 and Figure 3.
- Step 3: Based on the representative *RSSI* of the tag, the resident is in the room with the scanner with the maximum *RSSI* value.

**Figure 4:**
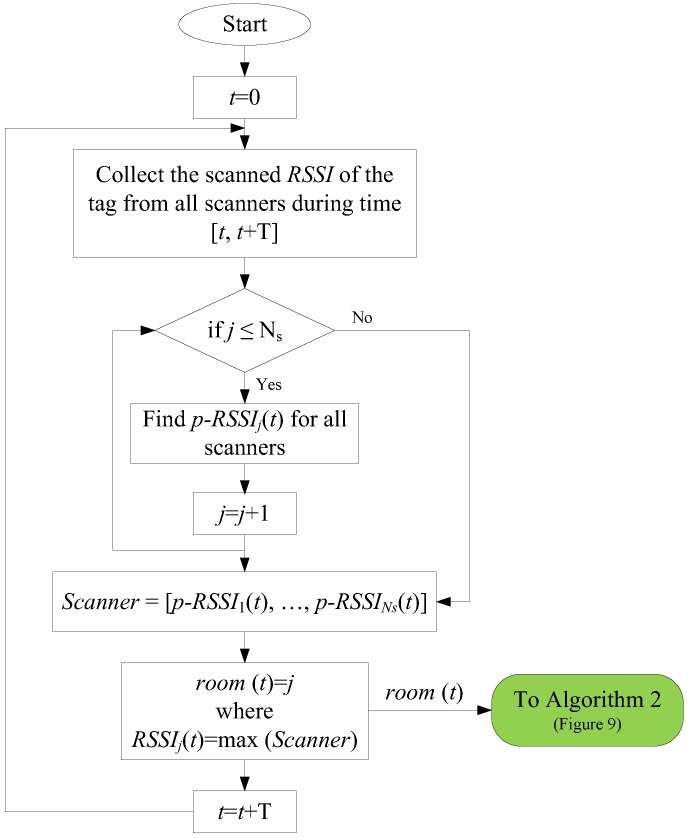
Flowchart of room-level localization in a smart home using raw BLE data (Algorithm 1).

The flowchart of localization using the *p*-*RSSI* index is shown in Figure 4.

## 3. Choose Optimal Advertising Parameters

**Figure 5:**
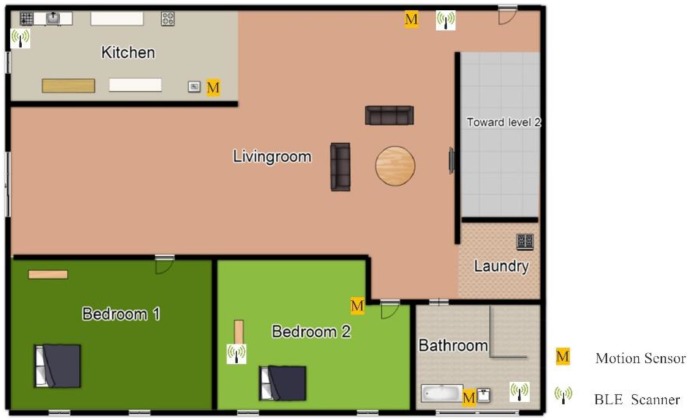
Single resident smart home architecture.

The set-up shown in Figure 1 is implemented in a home to examine the impact of advertising parameters on improving the localization accuracy. The architecture of the smart home is shown in Figure 5. The main area of this home monitored by sensors, includes Bedroom 2, Kitchen, Bathroom, and Living room. Four motion sensors are used as the activity sensors, while four BLE scanners are installed to localize the resident. As there is no difference between single- and multi-residency in this case, we only consider a single resident in this environment, to assess the impact of the advertising time interval and the advertising power level on localization accuracy.

### 3.1. Impact of the Advertising Time Interval

**Figure 6:**
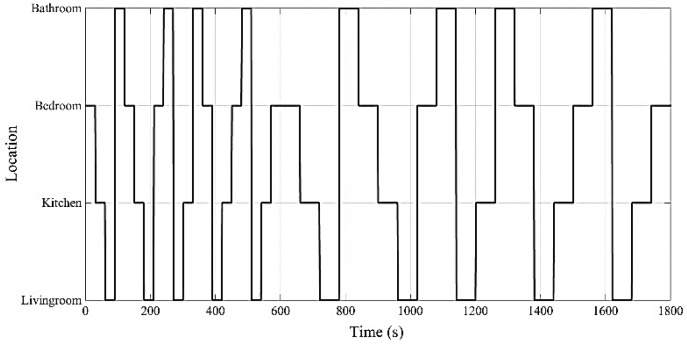
Ground truth path for the case study.

To study the impact of the advertising time interval on the accuracy of the localization, the ground truth path, shown in Figure 6, is used. As shown in this figure, two types of room change events are considered in this study: 20 events with a duration of 30 s in each room, and 20 events with a duration of 60 s in each room. In total, a 30 min ground truth path is followed by an individual. Four tags are programmed to advertise with specific time intervals, given in Table 1. Residents carrying all the tags (to have a unique test environment for all of the advertising time interval scenarios) are moving around the house based on the ground truth path.

**Figure 7:**
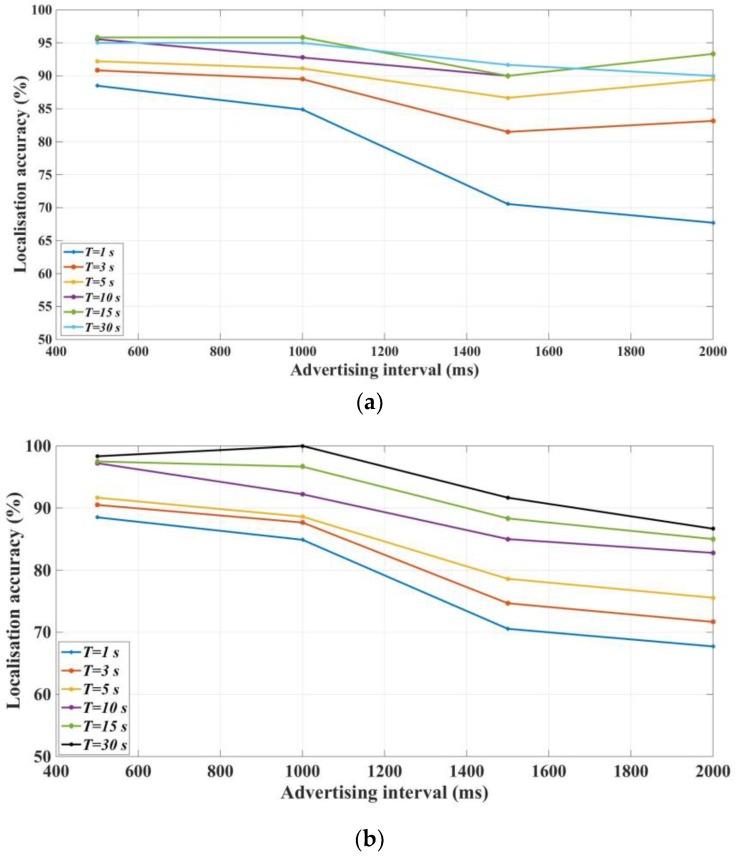
Impact of the advertising time interval on localization accuracy: (**a**) *p*-*RSSI* index; (**b**) *m*-*RSSI* index.

Figure 7 shows the localization accuracy using the two defined *RSSI* representatives. As noted above, the aim was to localize the resident at different *T* using two indices. Based on the provided results in Figure 7a, the following points can be concluded for the localization using the *p*-*RSSI* index:

- The localization accuracy is nearly constant for an advertising interval <1000 ms.
- The localization accuracy does not decline considerably when *T* is more than 5 s.
- The localization accuracy is maximized at *T* = 15 s.

Moreover, Figure 7b shows the results for the *m-RSSI* index. The following points can be extracted from this figure:

- The localization accuracy is nearly constant for an advertising interval <1000 ms.
- The localization accuracy declines considerably for advertising intervals more than 1000 ms.
- The localization accuracy increases when the time step increases.

By comparing the results of both indices, the parameters listed in Table 2 are proposed to have the highest accuracy.

It is interesting to notice that the *RSSI* index, due to its inherited nature as a radio signal, is not a stable measurement. That is why, in Figure 7a, we can see a small variation in accuracy when T = 15 s, which performs better than others while the advertising interval is between 1000 to 2000 ms. To mitigate the effects of instability of the *RSSI* signal, we propose using two types of measurements here, i.e., *m-RSSI* and *p-RSSI*. Specifically, since *m-RSSI* adopts mean *RSSI* values, its performance is more resistant to environmental changes, as shown in Figure 7b. On the other hand, *p-RSSI* may have better accuracy, but it is more vulnerable to influences from the surrounding environment.

### 3.2. Impact of the Advertising Power Level

In this case, tags are programmed to advertise with different power levels to achieve various broadcasting signal strength. Table 3 lists all of the power levels used in our tests, which are represented as decibel-milliwatts (dBm), the power ratio to one milliwatt (mW). Let *x* be the value of dBm, then the actual power, *P*, can be calculated as:

$$P=1\;\mathrm{mW}\times 10^{\frac{x}{10}}$$

Therefore, 0 dBm corresponds to 1 mW, 4 dBm means 2.5 mW, while −30 dBm means 0.001 mW. These settings affect how strongly the tag broadcasts its information. The advertising time interval is also set to 1000 ms for all tags.

**Figure 8:**
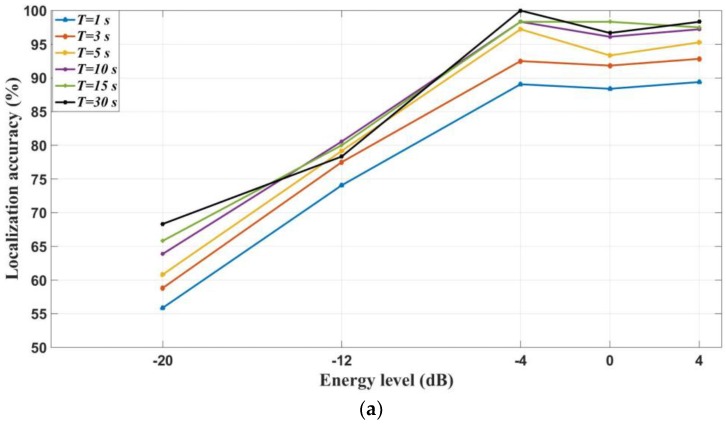

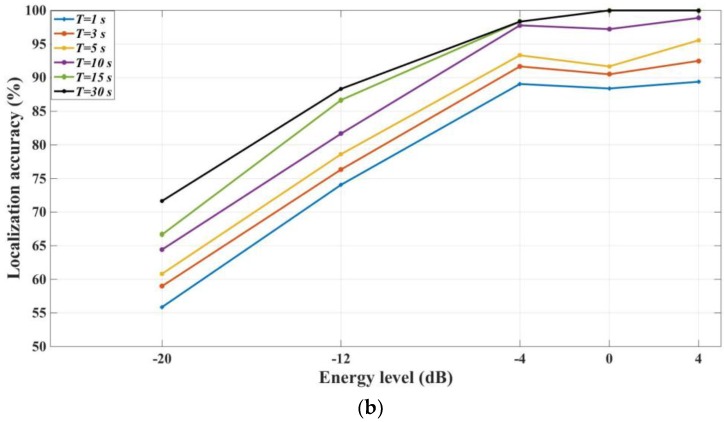
Impact of power level on localization accuracy: (**a**) *p*-*RSSI* index; (**b**) *m*-*RSSI* index.

The same ground truth path is followed to assess the performance of this approach for different advertising power levels. The results of localizing these tags using *p-RSSI* values are shown in Figure 8a. The following points can be extracted from the results:

- Power level 4 has better results for the *p*-*RSSI* index. This means that with a higher advertising power level, the fluctuation of *RSSI* caused by environments can be mitigated effectively.

The following points can also be extracted from the results in Figure 8b for the *m-RSSI* index:

- The accuracy is continuously increasing with the increasing of the advertising power level.
- The localization accuracy is maximized when *T* = 30 s.

It is interesting to note that although the authors of [23] claimed that Bluetooth has better performance for room-level accuracy—because of its lower advertising power level, compared to Wi-Fi technology—we observed in our experiments that, on the contrary, higher power settings are generally favored in terms of boosting localization accuracy. It can be seen that the higher power can improve the localization accuracy for the *m*-*RSSI* index. Therefore, it can be concluded that the advertising power level, which needs to be tuned properly, has an important impact on localization accuracy. The optimal advertising parameters for the proposed BLE localization are summarized in Table 4.

## 4. Integrating Activity Sensors to Improve Accuracy

**Figure 9:**
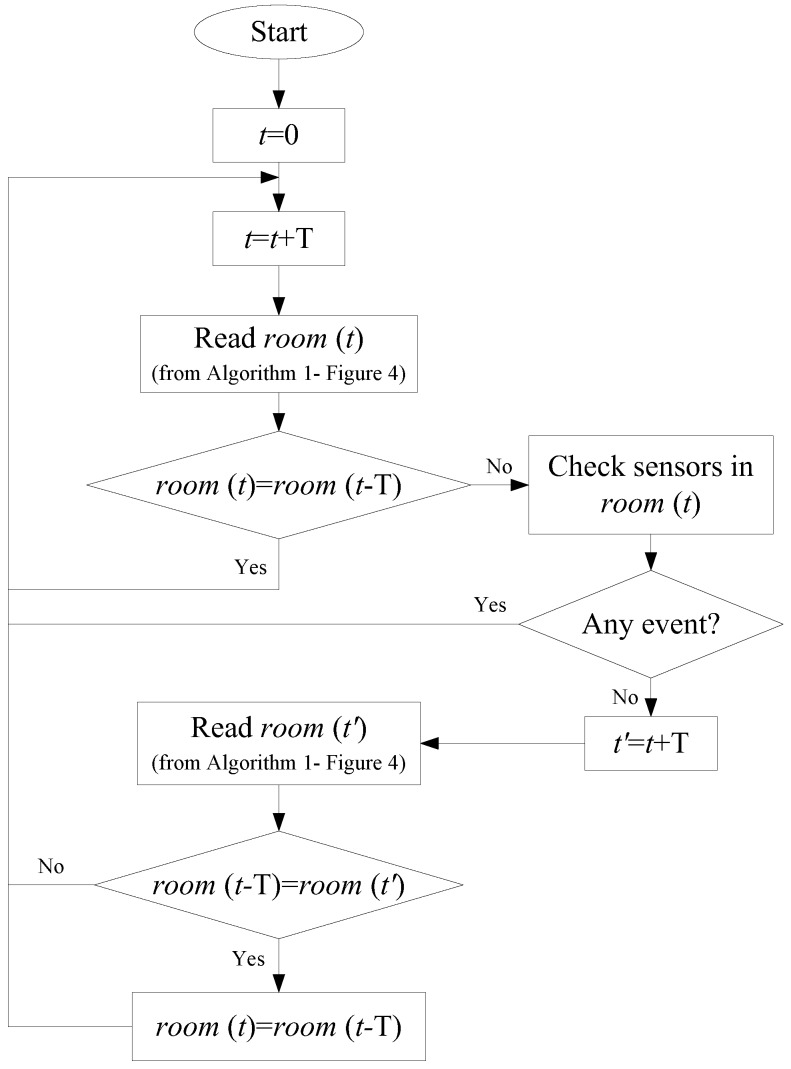
Flowchart of room-level localization in smart homes using BLE data and the activity sensor (Algorithm 2).

As the *RSSI* value is so sensitive to the environment, there may be some error in the localization. To improve the accuracy of the localization, a supplementary algorithm is proposed that uses the output of the previous algorithm and the data of activity sensors in smart homes. The flowchart is given in Figure 9. For each time step, if the detected room for the resident is different from the previous detected room, the event of the activity sensors in the new room is checked. If there is a new event during this time step, the new room is considered as the correct detection. However, if there is no event in the new room, the previous and next samples in the room output data are used to detect the correct room. Based on the experiment set-up in Figure 5, the results of applying this algorithm to two sample cases are shown in Table 5 and Table 6. These results, when compared with the results using Algorithm 1, show that the supplementary algorithm can improve the accuracy of the localization.

## 5. Multi-Residential Activity Labelling Using BLE Localization Technology

**Figure 10:**
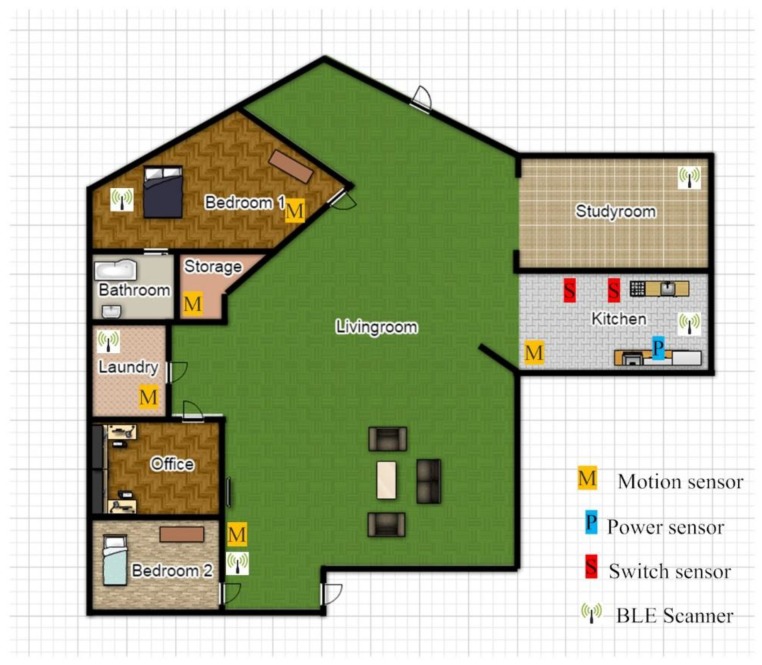
Multi-resident smart home architecture.

The proposed multi-residential localization approach has been trialed in a real smart home. There are two residents that are living in this home. The architecture of this smart home is shown in Figure 10. Three types of smart home sensors—including five motion sensors, two switch sensors, and one power sensor—are used to monitor the residents’ activities. Additionally, five BLE scanners are used to localize the residents in the main monitoring area. The main monitoring area includes a Kitchen, Study room, Living room, Laundry, and Bedroom 1, as residents spend most of their time in these areas. For the purpose of this paper, if residents use the Bathroom or Storage, then they will be considered to be in Bedroom 1. Moreover, presence in the Office and Bedroom 2 will be associated with the room with the closest scanner, which is the Living room in this case.

**Figure 11:**
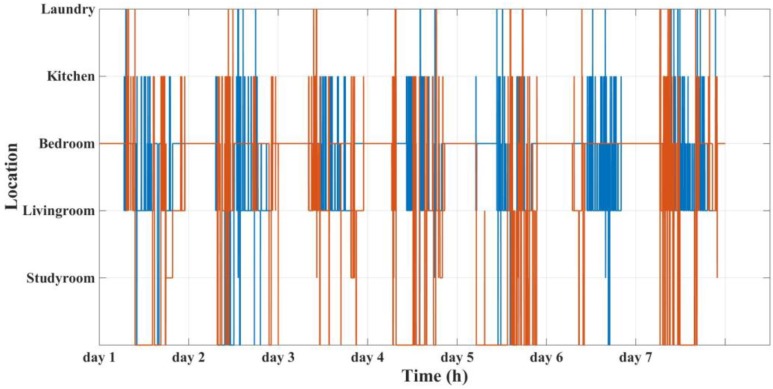
Residents’ localization results for the whole week.

The main aim of this trial is to study some of the main features of multi-residential activity labelling through BLE localization. The advertising interval for the tags is set to 1 s. The set-up is trialed for 1 week. The localization results for the whole week are shown in Figure 11. To see more details, three sample days are shown.

**Figure 12:**
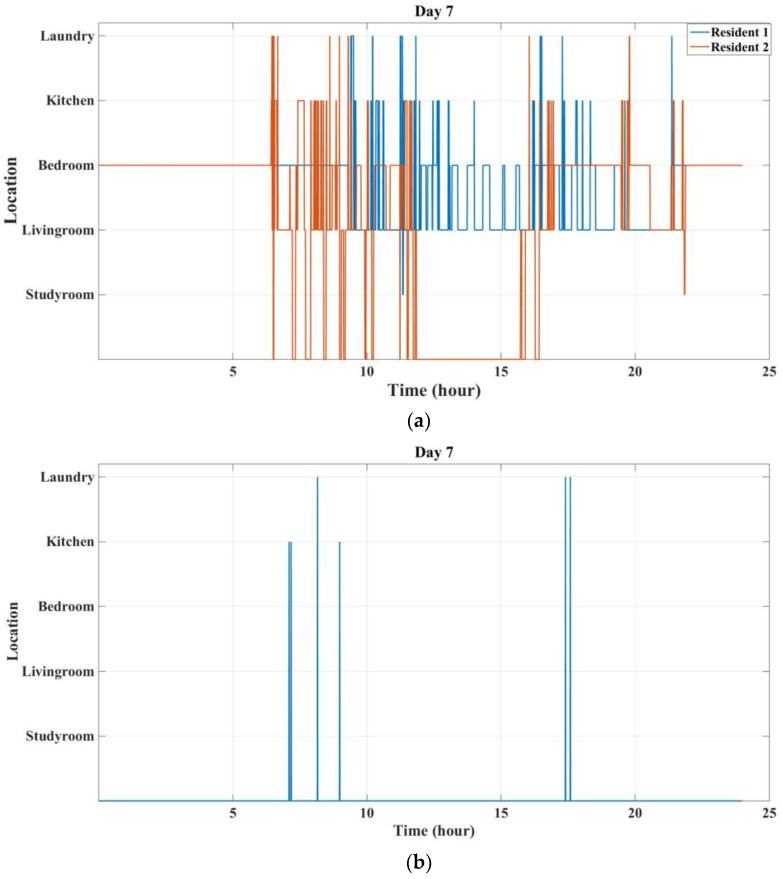
Localization and activity labelling results for the seventh day: (**a**) Resident localization results; (**b**) Non-labelled sensor events.

Figure 12 shows the results of localization and activity labelling for the residents on the seventh day. Figure 12a shows the localization results of the residents in different rooms during this day. It can be seen that resident 2 leaves Bedroom at 06:25 a.m., does some activities in the Laundry, Kitchen, and Living room, and then leaves the house at 11:50 a.m. While resident 1 wakes up at 09:17 a.m. and then stays in the home for the whole day. Based on the localization data, the events that are generated by the activity sensors are labelled using the individual’s location. Figure 12b shows those activities that cannot be labelled based on the localization data. Based on this figure, there are six sensor activities (out of 239 events during this day) that cannot be labelled by any resident. This is because we cannot be 100% accurate in localizing and labelling the activities, as the *RSSI* changes quickly or we may have missing *RSSI*. Also, these two residents are seniors and they will have community service providers that are working at their home from time to time. These “activities” may be caused by those caregivers and thus cannot be correctly labelled through *RSSI*. Based on these results, it can be said that the residents were carrying their tags during the whole day, as most of the sensor data can be labelled by the identity of the individuals.

**Figure 13:**
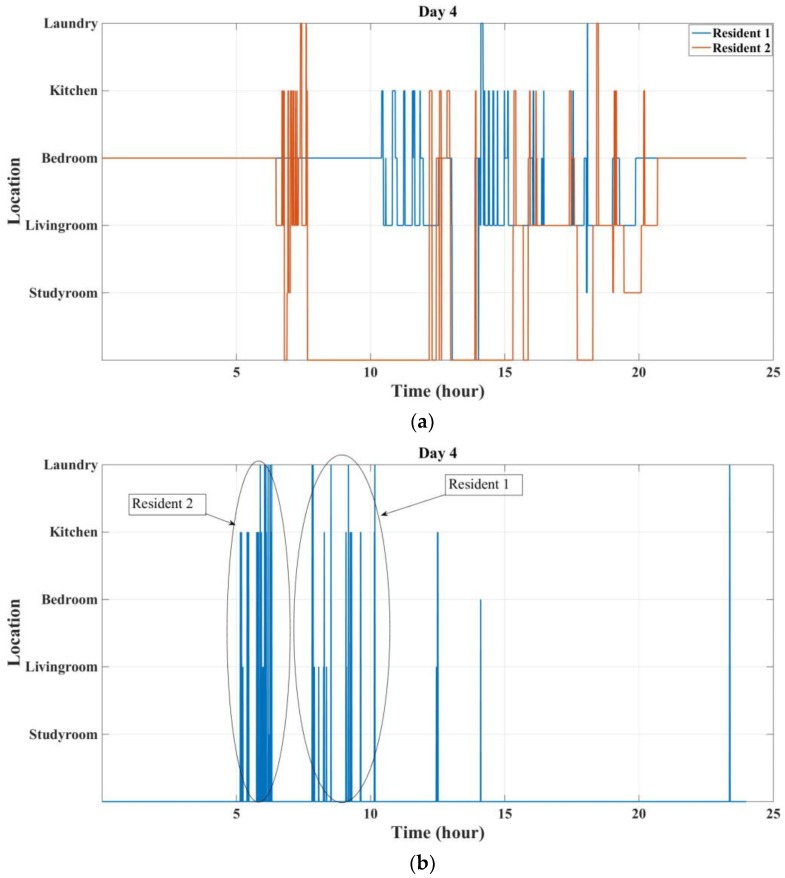
Localization and activity labelling results for the fourth day: (**a**) Residents localization results; (**b**) Non-labelled sensor events.

Figure 13 shows the results for the fourth day. It can be seen that there are a lot of sensor events that cannot be correctly labelled based on localization data. It can be predicted that residents take off their tags at night, while forgetting to wear them in the morning. For instance, based on the tag localization, it can be said that resident 2 left the Bedroom at 06:28 a.m. However, there are some events that occur before this time which can be considered as attributable to the resident of interest as the other resident usually wakes up around 10:00 a.m. The same thing is happening with the second resident, as there are some events that occur before the tag leaves the Bedroom (10:59 a.m.), while the other resident is not at home. It can be seen that these two sequences of events can be associated with the residents to improve the performance of this approach. In conclusion, it can be said that forgetting the tag can clearly impact the performance of sensor data labelling.

**Figure 14:**
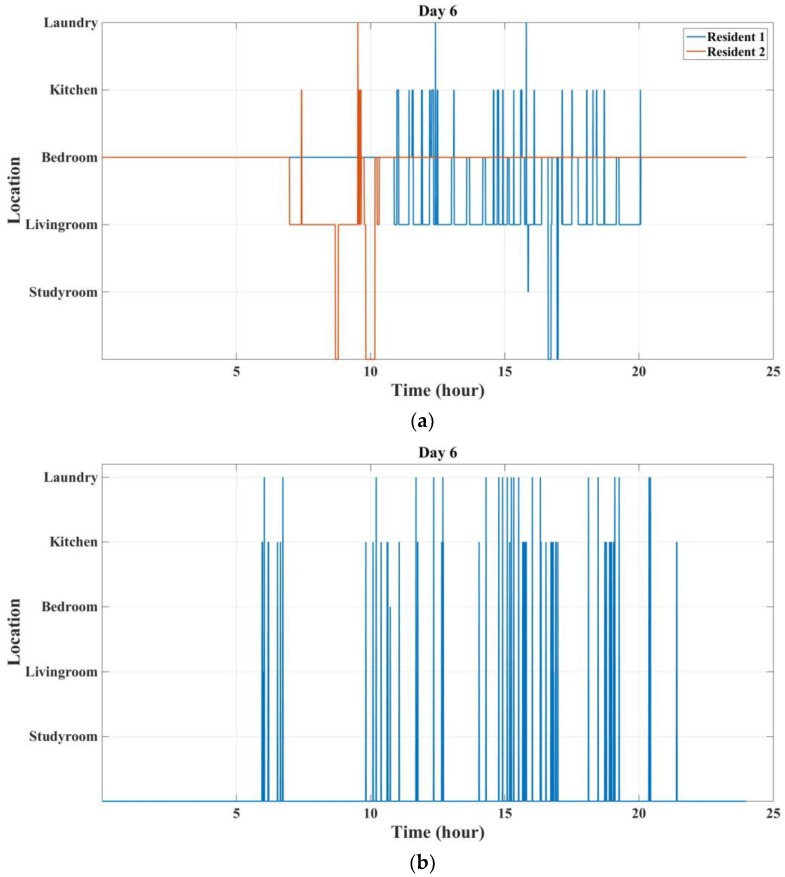
Localization and activity labelling for the sixth day: (**a**) Location of the resident; (**b**) Non-labelled sensor events.

Figure 14 shows a worst-case scenario, which is the sixth day. It can be seen that there are a lot of sensor events that cannot be labelled during this day. The missing events include two main parts. The first part is when resident 2 wakes up, which can be associated with this resident as the other resident is still asleep. However, it can be seen that after 10:00 a.m., resident 2 is in the Bedroom for the whole day. It can be said that this resident left the tag in Bedroom for the whole day. Therefore, there are a lot of activities that cannot be associated with the residents. In some cases (like the previous scenario), forgetting the tag can be detected and can be considered to improve the localization. However, in some cases, it is very difficult to associate the activities with the residents.

## 6. Conclusions

This paper studied the performance of BLE technology for the purpose of resident localization and activity labelling. We focused on improving room-level accuracy, as required by many smart home applications. The impacts of different localization parameters on the accuracy of this approach were studied. Additionally, an optimal tuning parameter was proposed to localize each resident, based on their tag *RSSI* value. The results with a ground truth path demonstrated promising outcomes for these algorithms. This localization method was also tested in a smart home with two residents, to automatically label their activities that were captured by the activity sensors.

We found that although BLE approaches can achieve high accuracy, forgetting the tag is always one of the main issues with this wearable approach, which greatly reduces the activity labelling accuracy. Also, in this paper, we assumed that no interfering signals, such as Wi-Fi from neighboring devices, coexist on the same frequency band. Investigation into the impact of these interferences is a potential area of research for future works.

## Figures and Tables

**Table 1:** Advertising time interval for tags.

Tag	Advertising Time Interval (ms)
1	500
2	1000
3	1500
4	2000

**Table 2:** Optimal advertising parameters.

Parameter	*p*-*RSSI* Index	*m*-*RSSI* Index
Time step	*T_opt_*_._ = 15 s	*T_opt_*_._ = max(*T*) = 30 s
Advertising interval	*T*_A, *opt.*_ = *T_S_* = 1 s	*T*_A, *opt.*_ = *T_S_* = 1 s

**Table 3:** Advertising power level for tags.

Tag	*E* (dBm)	Tag	*E* (dBm)
1	−20	4	0
2	−12	5	+4
3	−4	-	-

**Table 4:** Optimal parameters for the proposed localization approach.

*RSSI* Index	Time Step	Advertising Interval	Advertising Power Level
*m*-*RSSI*	max(*T*)	*T_S_*	max(*E*)

**Table 5:** Results of two algorithms (*T_A_* = 1 s; *E* = −4 dB; *RSSI* index = *m*-*RSSI* index).

Time Step	1	3	5	10	15	30
Localization accuracy with Algorithm 1	84.89	87.67	88.61	92.22	96.67	100
Localization accuracy with Algorithm 2	87.61	90.33	91.94	93.89	96.67	100

**Table 6:** Results of two algorithms (*T_A_* = 1 s; *E* = 4 dB; *RSSI* index = *m*-*RSSI* index).

Time Step	1	3	5	10	15	30
Localization accuracy with Algorithm 1	89.39	92.5	95.56	98.89	100	100
Localization accuracy with Algorithm 2	91.28	93.83	96.67	100	100	100
